# Remembrance of happy things past: positive autobiographical memories are intrinsically rewarding and valuable, but not in depression

**DOI:** 10.3389/fpsyg.2015.00222

**Published:** 2015-03-03

**Authors:** Chong Chen, Taiki Takahashi, Si Yang

**Affiliations:** ^1^Department of Psychiatry, Hokkaido University Graduate School of MedicineSapporo, Japan; ^2^Department of Behavioral Science, Center for Experimental Research in Social Sciences, Hokkaido UniversitySapporo, Japan; ^3^Department of Psychology, University of Rhode IslandKingston, RI, USA

**Keywords:** autobiographical memory, positive emotion, reward, value, striatum, medial prefrontal cortex, resiliency, depression

Recent evidence suggests that retrieving positive memories increases positive emotion and achieves a mood regulation effect, likely by activating the brain reward circuit (Speer et al., 2014). Here we argue that retrieving positive memories may be a useful strategy in everyday mood regulation for healthy people, but not for people with depression.

## Intentional activities to boost positive emotions

Positive emotion remains a core topic within positive psychology (Fredrickson, 2001, 2013). Itself a marker of the optimal well-being, positive emotion not only undoes negative emotions, but also broadens positive thinking and activities, builds invaluable resources and relationships, and therefore ultimately promotes further positive emotions (Fredrickson, 2001, 2013). Fortunately, beyond comparatively unmodifiable genetic and environmental factors, people can actively engage in many intentional activities to increase their positive emotions (Lyubomirsky et al., 2005). For instance, pursuing self-concordant goals (Sheldon, 2014), expressing gratitude (Seligman, 2011), counting one's blessings (Lyubomirsky et al., 2005), and retrieving positive memories (Rusting and DeHart, 2000; Joormann and Siemer, 2004; Duckworth et al., 2005; Joormann et al., 2007; for many other activities, see Ben-Shahar T 2010 *Even happier: a gratitude journal for daily joy and lasting fulfillment*. New York: McGraw-Hill).

Notably, amongst these intentional activities, retrieving positive memories has been gaining more and more research interest. Traditionally this strategy was employed to induce positive emotions in psychological experiments (Rusting and DeHart, 2000). It has only recently been suggested that this strategy may be useful in mood regulation (Joormann and Siemer, 2004; Duckworth et al., 2005; Joormann et al., 2007). A recent study (Speer et al., 2014) further justified this effectiveness by uncovering the underlying neural mechanism.

## Positive autobiographical memories are intrinsically rewarding: the neural mechanism

Speer et al. (2014) asked subjects to perform a cue recall task while under functional magnetic resonance imaging (fMRI) scanning. In the task, subjects had to recall autobiographical memories upon being presented positive (e.g., family vacation, visiting Disneyland) and neutral (e.g., packing for a trip) life event cues. Consistent with previous research, Speer et al. (2014) found that retrieving positive memories induced greater emotional intensity and more positive feelings than neutral memories. Accompanying the positive memories, the reward circuits i.e., the corticostriatal network, in particular striatum (caudate), ventromedial prefrontal cortex (PFC), orbitofrontal cortex and anterior cingulate showed significantly increased activity. Moreover, subjective rating of positive emotion was significantly correlated with activity in caudate and medial PFC. As the magnitude of positive emotion increased, activity in caudate and medial PFC also increased. In the end, Speer et al. (2014) examined whether the positive feelings induced positive memories could change the basal mood. They found that, though not everyone who tried to recall positive memories had improved mood, those who had improved mood after retrieving positive memories did demonstrate significantly increased activity in the right ventral striatum and left putamen (dorsolateral striatum). In contrast, those subjects who failed to show more positive mood following retrieving positive memories did not demonstrate these increased striatal activity. This suggests that striatal activation may be a prerequisite to improve mood.

Extensive evidence from computational research of reinforcement learning has established that both caudate and medial PFC are responsible for coding reward value and signaling reward prediction error, which are essential for reinforcement learning (Daw et al., 2011; O'Doherty, 2011; Lee et al., 2012; Liljeholm and O'Doherty, 2012). Therefore, it can be speculated that retrieving positive memories, which generates a positive reward prediction error, is intrinsically rewarding. Further, the activation of the reward circuits especially caudate and medial PFC might be the underlying neural mechanism by which it achieves its effect on mood regulation.

Interestingly, after the cue recall task, Speer et al. (2014) further asked subjects to perform a monetary reward game. A contrast of gains and losses revealed significant activation in the ventral striatum, which also showed greater activation to the positive memories in the same individuals. This finding suggests that retrieving positive memories involves the similar neural substrate as tangible and extrinsic monetary rewards.

## Positive autobiographical memories in depression

Given its usefulness in mood regulation, it is tempting to ask whether this strategy would be effective in treating depression or major depressive disorder clinically.

The work of Joormann and colleagues provided initial evidence that the answer to this question might be negative (Joormann and Siemer, 2004; Joormann et al., 2007). Although retrieving happy memories repaired sad mood in non-dysphoric or never-depressed subjects, it left dysphoric or previously depressed subjects' sad mood unchanged, and made currently-depressed subjects' sad mood even worse (Joormann and Siemer, 2004; Joormann et al., 2007). Joormann and colleagues proposed that recalling happy memories in depressed subjects might trigger the self-focused negative rumination in depressed subjects. As a result, the initial positive feelings induced by positive memories are compared to the depressive feelings which are generated by rumination. This comparison consequently worsens the basal mood (Joormann and Siemer, 2004; Joormann et al., 2007). We agree with their reasoning and would like to elaborate and extend their interpretation in the following.

Firstly, the process of retrieving positive memories for depressed people is effortful, which may diminish the reward value of the actually recalled positive memories. Depressed people have impaired memory for positive stimuli but preferential memory for negative stimuli (Mathews and MacLeod, 2005; Gotlib and Joormann, 2010; Belzung et al., 2014). They recall positive stimuli much slower than negative stimuli (Lloyd and Lishman, 1975), and recall less positive stimuli than negative stimuli (Matt et al., 1992). They also recall less positive stimuli (Matt et al., 1992) as well as stimuli that has been previously associated with reward (Dillon et al., 2014) than healthy controls. In contrast, their negative memories occur habitually and induce the vicious circle of rumination (Watkins and Nolen-Hoeksema, 2014). In the meantime, they also have difficulty in removing irrelevant emotional material from their working memory (Yoon et al., 2014). These memory deficits might be related to abnormal activation in the medial PFC, hippocampus, amygdala and dopaminergic midbrain (Arnold et al., 2011; Dillon et al., 2014). From the viewpoint of feelings-as-information theory (Schwarz, 2012), besides the contents of information, people also use the fluency of information processing (e.g., retrieval) itself as a source of information. Specifically, it is natural for people to prefer information that is easy to perceive, process, or recall, but to consider information that is difficult to perceive, process, or recall as untrue, unreliable, and unimportant. Consequently depressed people may conclude that their habitual negative memories are more real and that their positive memories, which are effortful for them to retrieve, are actually not rewarding and valuable. Since effortful retrieval, as compared to automatic retrieval, might be considered a kind of loss of control, this account fits well with recent finding that perceived control, in the form of having the opportunity to choose, is rewarding and can activate the reward circuitry (Leotti and Delgado, 2014). Therefore, loss of control over the process of retrieving positive memories may diminish the reward value of the recalled positive memories.

Secondly and more fundamentally, even if depressed people can successfully recall positive memories (for instance, with support from others or by memory supporting techniques), their dysfunctional reward circuitry may inhibit them from up-regulating positive moods. The core feature of depression is anhedonia, the inability to experience positive experiences (American Psychiatric Association, 2014). At the neural level, depression is characterized by impaired reward learning and dysfunctional corticostriatal circuits (Russo and Nestler, 2013; Chen et al., under revision). Further, fMRI research has consistently found low striatal responses to rewarding outcomes in people with depression (Groenewold et al., 2013; Zhang et al., 2013). Therefore, in the context of the finding by Speer et al. (2014) that striatal reactivation to positive memories is a prerequisite for up-regulation positive mood, it is reasonable to assume that, even if depressed people can retrieve positive memories, these memories may not activate the reward circuits and increase positive mood. In support of this, research has been done to examine the effect of resiliency, an important protective factor of depression (Beardslee et al., 2012), on the neural reactions to retrieving positive memories. It was found that subjects' self-reported resiliency was positively associated with the activity in right caudate during the recall of positive memories (*r* = 0.458, *p* = 0.05) (Speer et al., 2014). Subjects with higher self-reported resiliency tended to have higher caudate response to positive memories. Namely, the reward circuits especially the caudate might be more readily activated during everyday life in resilient individuals, which induces more positive emotions and positive mood (Duckworth et al., 2005). This is equally to say that, less resilient people or people at high risk for depression may have deficits in the activation of caudate during recall of positive memories, and that they fail to show sustained positive mood and gradually develop depression.

Still, another possibility exists (We thank the reviewer for suggesting this possibility). Due to the dysfunctional reward circuitry in depression, those “positive” memories may not be perceived as positive in the first place. That is, they may not induce positive feelings, not to speak of changing the basal depressive mood (Speer et al., 2014).

These findings together support the hypothesis that retrieving positive memories might not be intrinsically rewarding for depressed people. In an experiment, Speer et al. (2014) visited this hypothesis by examining if and to what extent subjects were willing to give up money in order to retrieve a positive memory. Here each positive or neutral memory cue was accompanied by a monetary payoff (ranging from 1 to 4 cents). For instance, subjects could receive 2 cents to recall family vacation (positive) but 4 cents to recall packing for a trip (neutral). Therefore, subjects had to forgo 2 cents to recall family vacation instead of packing for a trip. This 2 cents was called the point of subjective equity (PSE), which reflects the subjective value of choosing a positive vs. neutral memory. In this way, the value of retrieving positive memories was monetarily quantified. Speer et al. (2014) found that, on choices of equal value, subjects chose positive memories 85% of the time, whereas on all other choices, subjects chose positive memories 70.7% of the time. On average, subjects forwent 1.94 cents to retrieve a positive rather than negative memory. It suggested that subjects did prefer positive memories and were willing to give up monetary payoff to retrieve them. In contrast, this was not the case in depression. The authors found that, there was a significant correlation between PSE and depressive symptoms (measured by Beck depression inventory; *r* = 0.39, *p* = 0.047). Subjects with more severe depression symptoms had higher PSE. In other words, their positive memories were less valuable compared to tangible money and therefore they were less willing to forgo money to recall a positive memory.

We have previously demonstrated that depressed patients show altered temporal discounting of future reward and punishment (Takahashi et al., 2008). The above finding by Speer et al. (2014) demonstrated that it is likely that the impaired temporal discounting also extends toward the past (Yi et al., 2006) (i.e., rewards from the past may be greater discounted in depression). This is in line with the proposal in computational research that patients with depression might have reduced memory of previous reward reinforcement (Dombrovski et al., 2010; Chen et al., under revision). These deficits are likely to share the common underlying pathophysiology, the dysfunctional corticostriatal reward circuits (Russo and Nestler, 2013; Chen et al., under revision).

## Conclusion

Available evidence suggests that retrieving positive autographical memories is intrinsically rewarding and valuable. Positive memories evoke brain reward reactions similar to those by tangible and extrinsic monetary rewards. People are willing to forgo more tangible monetary payoffs to retrieve positive memories. However, this may not be the case for depressed people. Patients with depression have difficulty in retrieving positive memories. More fundamentally, in face of positive memories, their dysfunctional reward circuitry may inhibit them from up-regulating positive moods. Or, due to the dysfunctional reward circuitry, those “positive” memories may not be perceived as positive in the first place. Thus, we argue that retrieving positive autographical memories may be an effective strategy in everyday mood regulation for healthy people, but to achieve its therapeutic antidepressant effect clinically, overriding the dysfunctional reward circuitry especially the striatum may be the first step.

### Conflict of interest statement

The authors declare that the research was conducted in the absence of any commercial or financial relationships that could be construed as a potential conflict of interest.
